# Recent Progress in Two-Dimensional MoTe_2_ Hetero-Phase Homojunctions

**DOI:** 10.3390/nano12010110

**Published:** 2021-12-30

**Authors:** Jing Guo, Kai Liu

**Affiliations:** State Key Laboratory of New Ceramics and Fine Processing, School of Materials Science and Engineering, Tsinghua University, Beijing 100084, China; guoj_0@163.com

**Keywords:** two-dimensional materials, MoTe_2_, phase transition, homojunctions

## Abstract

With the demand for low contact resistance and a clean interface in high-performance field-effect transistors, two-dimensional (2D) hetero-phase homojunctions, which comprise a semiconducting phase of a material as the channel and a metallic phase of the material as electrodes, have attracted growing attention in recent years. In particular, MoTe_2_ exhibits intriguing properties and its phase is easily altered from semiconducting 2H to metallic 1T′ and vice versa, owing to the extremely small energy barrier between these two phases. MoTe_2_ thus finds potential applications in electronics as a representative 2D material with multiple phases. In this review, we briefly summarize recent progress in 2D MoTe_2_ hetero-phase homojunctions. We first introduce the properties of the diverse phases of MoTe_2_, demonstrate the approaches to the construction of 2D MoTe_2_ hetero-phase homojunctions, and then show the applications of the homojunctions. Lastly, we discuss the prospects and challenges in this research field.

## 1. Introduction

Over the past few years, the complexity of integrated circuits (IC) in the semiconductor industry has increased with the decrease in the size of components [1,2]. However, traditional silicon-based transistors have been confronted with fundamental limits induced by quantum mechanics and thermodynamics at the nanometer scale [3], thereby leading to several problems, such as the short-channel effect [4,5] and severe carrier scattering by surface dangling bonds [6,7], which would degrade the device performance and hinder the scaling. To solve these problems, low-dimensional electronic materials including transition metal dichalcogenides (TMDs) have been intensively studied due to their prominent advantages, including atomically thin thickness without dangling bonds, diversity of bandgaps, and excellent performance that is superior to their silicon counterparts [8]. For an ideal 2D transistor, there are four essential elements, which are a high-mobility 2D semiconductor channel, a high-κ dielectric, an ultrasmooth substrate with high thermal conductance, and ohmic contacts with low Schottky barrier height (SBH) [9] and low contact resistance [8]. Thus far, it remains challenging to realize ohmic contacts with ultralow SBH [10] because of the large energy difference between the semiconductor electron affinity and the metal work function, as well as Fermi-level pinning resulting from metal-induced gap states (MIGS) [11,12,13]. Moreover, in the conventional device fabrication process involving multi-step procedures, 2D electronic devices are vulnerable to defects and impurities, leading to a deterioration in their performance.

**Figure 1:**
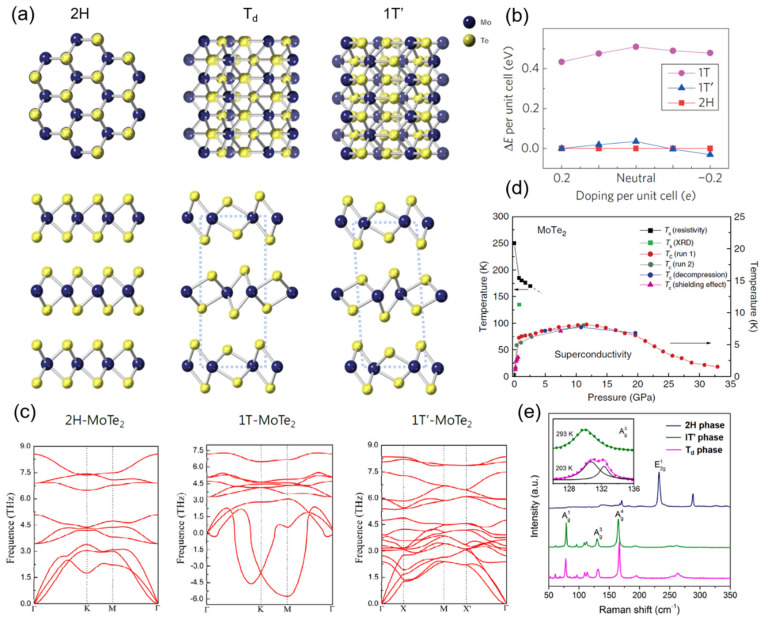
Structures and properties of different phases of MoTe_2_. (**a**) Atomic models of 2H-, T_d_-, and 1T′-MoTe_2_; (**b**) relative total energy per unit cell for 1T-, 1T′-, and 2H-MoTe_2_ with different electron doping levels (**c**) phonon dispersion curves of 2H-, 1T-, and 1T′-MoTe_2_, Reprinted with permission from [17] 2015 Springer Nature.; (**d**) electronic phase diagram of MoTe_2_ extracted from various measurements, Reprinted with permission from [24] 2016 Springer Nature; (**e**) Raman spectra of 2H-, 1T′-, and T_d_-MoTe_2,_ Reprinted with permission from [18] 2019 American Chemical Society.

Two-dimensional hetero-phase homojunctions provide a solution to these problems. Multiple phases of some TMDs [14] allow us to construct circuits composed of the metallic phase as electrodes or interconnects and the semiconducting phase as channels, which avoids external metal contacts and unnecessary film depositions [15]. Moreover, with careful selection of the appropriate materials, the SBH between the hetero-phases is expected to be ultralow if the interface is covalently bonded and atomically coherent [16]. Among all TMDs, the energy barrier between 2H- and 1T′-MoTe_2_ is extremely small (Figure 1b) [17], the moduli of these phases are also very close [18,19,20], and thus it is easy to alter the phase of MoTe_2_ from 2H to 1T′ and vice versa. This makes MoTe_2_ a perfect platform for the construction of 2D hetero-phase homojunctions. More interestingly, the 1T′ and T_d_ phases of MoTe_2_ possess some fascinating properties, such as topological insulator [21,22,23], superconductor [24], and Type-II Wyle semimetal (WSM) [25,26,27] properties. In recent years, MoTe_2_ hetero-phase homojunctions have been intensively studied due to the aforementioned advantages.

In this review, we summarize the recent progress in 2D MoTe_2_ hetero-phase homojunctions. First, we introduce the properties of three phases of MoTe_2_, namely the 2H, 1T′, and T_d_ phases [28]. Second, the strategies for constructing 2D MoTe_2_ hetero-phase homojunctions are demonstrated regarding two aspects, direct synthesis and post-processing. Next, we manifest applications of 2D MoTe_2_ hetero-phase homojunctions in electronics, optoelectronics, and catalysis. Finally, we discuss the current challenges and opportunities in this research field.

## 2. Phases and Properties of MoTe_2_

Near room temperature, MoTe_2_ exhibits three stable phases: 2H, 1T′, and T_d_ phases. These phases have different lattice structures, band structures, and fingerprints in optical spectra.

### 2.1. 2H Phase

As shown in Figure 1a, 2H-MoTe_2_ (α-phase) exhibits a hexagonal structure, and it belongs to the space group *P*6_3_/*mmc* with a honeycomb-like in-plane structure [28]. In addition, 2H-MoTe_2_ is an indirect bandgap semiconductor (bandgap 0.8 eV [29]) in bulk and few-layer states (Figure 1c). When thinned to a monolayer, however, it becomes a direct bandgap semiconductor (bandgap 1.1 eV [30]). Generally, 2H-MoTe_2_ is relatively stable in ambient conditions, but, due to the extremely small energy barrier with 1T′-MoTe_2_, it will convert into the 1T′ phase under excess Te deficiency or strain [31,32].

### 2.2. 1T′ Phase

Firstly, 1T′-MoTe_2_ (β-phase) has a monolithic structure, belonging to space group *P*2_1_/*m*, with a distorted centered honeycomb in-plane structure and slightly inclined interplane structure (β angle = 93°55′) [28], as shown in Figure 1a. Compared to 2H-MoTe_2_, 1T′-MoTe_2_ is more vulnerable to oxidation [33]. In addition, 1T′-MoTe_2_ is generally acknowledged to be metallic, whether in the bulk or monolayer state. However, there are some reports designating few-layer 1T′-MoTe_2_ as a semiconductor. Keum et al. reported that few-layer 1T′-MoTe_2_ exhibited a bandgap opening up to 60 meV (Figure 1c) and proposed that it was induced by strong spin–orbit coupling (SOC) [17]. More recently, tri-layer 1T′-MoTe_2_ was reported to possess a narrow bandgap of 28 meV, but, in thicker MoTe_2_ samples (>4 nm), such bandgap opening was not observed [34]. On the contrary, it was also reported that tri-layer 1T′-MoTe_2_ remained free of a bandgap [35]. It is still under debate whether few-layer 1T′-MoTe_2_ has a bandgap.

In addition, 1T′-MoTe_2_ was reported to be a topological insulator as well as a room-temperature ferroelectric material. A weak antilocalization effect was observed in monolayer 1T′-MoTe_2_, indicating the presence of strong SOC, which is related to topological insulator materials [22]. Bulk 1T′-MoTe_2_ was predicted to be a Z4-nontrivial higher-order TI (HOTI) driven by double band inversion [23], which has not been experimentally confirmed yet. Recently, robust room-temperature out-of-plane ferroelectricity was observed in monolayer distorted 1T-MoTe_2_ [36], which has sparked the exploration of fundamental physics and promising applications at the monolayer limit.

### 2.3. T_d_ Phase

T_d_-MoTe_2_ (γ-phase) has an orthogonal structure, belonging to space group *Pmn*2_1_ [28]. Compared to the 1T′ phase, the T_d_ phase displays a very similar but more regular structure (Figure 1a). From the cross-section direction, atoms in different layers of T_d_-MoTe_2_ are in good alignment with each other, whereas, in 1T′-MoTe_2_ atoms, layers slide a short distance and form a 0.94° tilt angle. Therefore, it is almost impossible to distinguish 1T′ from T_d_ MoTe_2_ at the level of the monolayer.

When the temperature is increased above ~250 K, the T_d_ phase is supposed to convert to the 1T′ phase [37]. In order to obtain stable T_d_-MoTe_2_ at room temperature, tungsten substitutional doping [38] and thinning the material to be below 10 nm [37,39] are effective strategies. Owing to its broken inversion symmetry, the T_d_ phase possesses abundant physical properties, such as superconductivity and Type-II WSM.

T_d_-MoTe_2_ exhibits pressure- and thickness-dependent superconductivity. The transition temperature T_c_ of T_d_-MoTe_2_ increases with higher pressure and smaller thickness. The T_c_ of bulk T_d_-MoTe_2_ was reported to be 0.10 K (Figure 1d) [24]. Guguchia et al. observed the two-gap s-wave symmetry of the superconducting order parameter in T_d_-MoTe_2_, and suggested a higher possibility of a topologically non-trivial s^+−^ state in T_d_-MoTe_2_ [40]. A fast-mode oscillation of the critical current I_c_ versus magnetic field B along the edge was observed in T_d_-MoTe_2_ by Wang et al., which was generated by fluxoid quantization and indicated the existence of a robust edge supercurrent [41]. As the breaking of the time-reversal symmetry or inversion symmetry is the prerequisite for WSM, T_d_-MoTe_2_ was predicted as a Type-II WSM due to its noncentrosymmetric lattice structure [26]. Kaminski et al. identified Fermi arcs, Weyl points, and novel surface states in bulk T_d_-MoTe_2_ with the angle-resolved photoemission spectroscopy (ARPES) technique, providing experimental evidence for T_d_-MoTe_2_ as a Type-II WSM [42]. Large magnetoresistance, a chiral-anomaly-related phenomenon, was reported in T_d_-MoTe_2_, further proving that T_d_-MoTe_2_ is a representative Type-II WSM [43,44].

## 3. Construction of 2D MoTe_2_ Hetero-Phase Homojunctions

### 3.1. Direct Synthesis

Chemical vapor deposition (CVD) is a universal and convenient method for the direct synthesis of 2D materials and heterojunctions, which is also viable in the case of MoTe_2_ hetero-phase homojunctions. The key to constructing 2D MoTe_2_ hetero-phase homojunctions is precise control of phases. The final phase of MoTe_2_ after growth is determined by several factors, which are growth temperature, growth atmosphere, and Te vacancy concentration.

**Figure 2:**
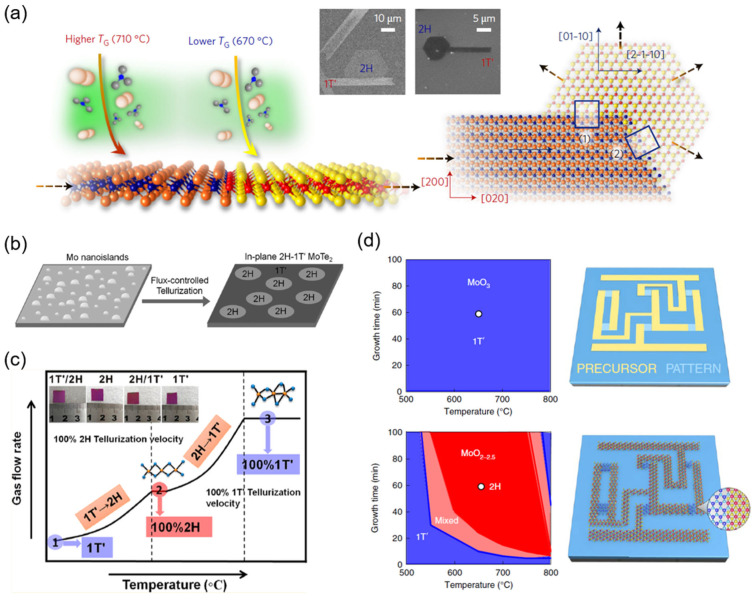
Direct synthesis of MoTe_2_ hetero-phase homojunctions. (**a**) Controlling growth temperature, Reprinted with permission from [16] 2017 Springer Nature; (**b**) flux-controlled tellurization Reprinted with permission from [45] 2017 John Wiley and Sons; (**c**) controlling growth temperature and gas flow rate Reprinted with permission from [46] 2017 American Chemical Society; (**d**) simultaneous tellurization of patterned precursors with different compositions, Reprinted with permission from [15] 2019 Springer Nature.

It was reported that the most thermodynamically stable phase varied under different processing temperatures [45]. Thus, by controlling the growth temperature, a certain phase can be selectively obtained. Sung et al. reported that the 1T′-phase was the dominant product at a higher temperature (710 °C), and the 2H phase dominated at a lower temperature (670 °C) [16]. By sequential growth of 1T′ and 2H-MoTe_2_, they obtained isolated lateral 1T′-2H-MoTe_2_ homojunctions (Figure 2a). With increasing temperature, Yang et al. observed the phase evolution shown in Figure 2c: pure 1T′-phase→1T′- and 2H-phase→pure 2H-phase→2H- and 1T′-phase→pure 1T′-phase [46].

Besides the growth temperature, the growth atmosphere plays an important role in controlling the phases in MoTe_2_. By changing the flow rate of either the inert carrier gas (N_2_) or active carrier gas (H_2_), phase evolutions with a tendency similar to the aforementioned were reported [46,47]. Such phase evolution was attributed to the tellurization velocity, and conclusions were drawn that a fast tellurization velocity favored 1T′ phase formation, while a moderate tellurization velocity favored the 2H phase [46]. It is possibly analogous to the formation of dendrites, wherein a fast cooling rate leads to plenty of dendrites, which possess a more disordered and defect-rich structure, while a moderate cooling rate results in a more regular and thermodynamically stable structure.

In essence, the process of phase transition in MoTe_2_ is structural transformation [48], which can be triggered by either external stimuli, such as temperature and growth rate, or internal factors such as strain and Te deficiency. The influence of strain in the phase control of MoTe_2_ will be discussed in the next section. The utility of the Te deficiency in the phase control of MoTe_2_ has been intensively studied. It was reported that the 1T′ phase of MoTe_2_ was more stable than the 2H phase when the Te deficiency exceeded 2%, which was confirmed by density functional theory (DFT) calculations [48]. In CVD synthesis, the concentration of Te deficiency is tunable by controlling the concentrations or compositions of precursors.

Strategies such as prolonging the growth time [49], increasing Te atomic flux by raising the source temperature (Figure 2b) [50], and controlling the concentration of MoO_3_ precursor by means of molecular sieves and overlapped substrates [51] were proven to be effective to provide excessive Te during the growth period and thereby obtain the stable stoichiometric 2H phase. Accordingly, the reverse phase transition from 2H to 1T′-MoTe_2_ could be realized by further annealing under a lower partial pressure of Te at the same temperature, followed by rapid quenching [49]. Moreover, a mixed-dimensional in-plane 1D–2D Mo_6_Te_6_–MoTe_2_ heterostructure was directly synthesized under medium Te flux [52].

By tellurizing precursors with different compositions, 2H and 1T′-MoTe_2_ were simultaneously synthesized by Zhang et al. [15]. The products after tellurizing MoO_2.0–2.5_ and MoO_3_ thin films were identified as 2H and 1T′-MoTe_2_, respectively (Figure 2d). The authors proposed that the selective phase synthesis was realized by the varied Te monovacancies densities in the products. Combining the above synthesis strategies with lithographic processes, 2D MoTe_2_ hetero-phase junctions [50,53] and their derivatives, such as MoTe_2_ field-effect transistor (FET) arrays [54,55] and integrated MoTe_2_ circuits [15], were successfully fabricated.

Recently, Xu et al. showed that the structure of 2H-MoTe_2_ could spread into the adjacent 1T′ phase under a Te-rich atmosphere [56]. Triggered by an implanted single-seed crystal, they synthesized wafer-scale single-crystalline 2H-MoTe_2_ via 2D epitaxy of polycrystalline 1T′-MoTe_2_.

### 3.2. Post-Processing

Besides direct synthesis, post-processing is also a very versatile strategy to realize 2D MoTe_2_ hetero-phase homojunctions. Owing to the small energy barrier between the 2H and 1T′ phases, external stimuli, such as a laser, electrostatic gating, mechanical deformations, chalcogen alloying, and lithium-ion (Li^+^) intercalation, can easily induce phase transition in MoTe_2_.

**Figure 3:**
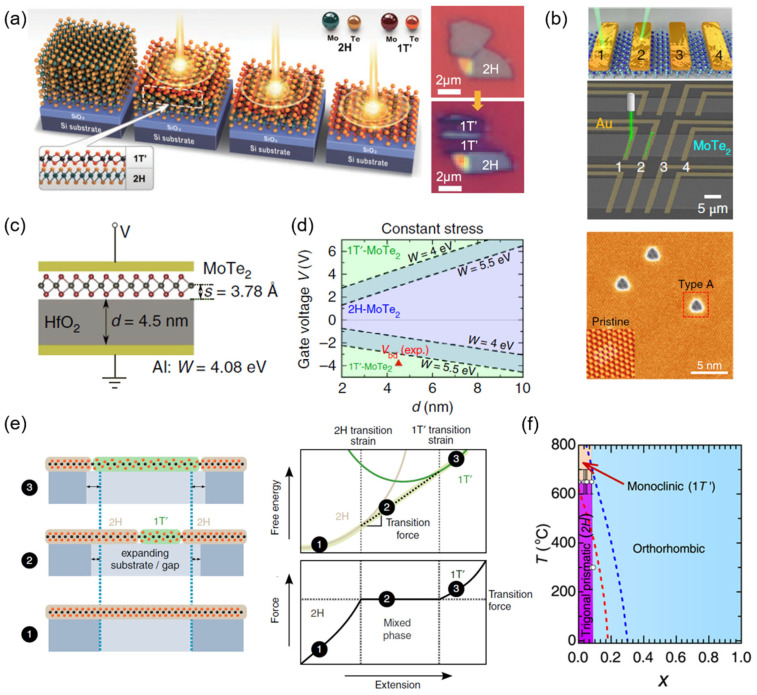
Construction of MoTe_2_ hetero-phase homojunctions via post-processing. (**a**) Laser-induced phase patterning, Reprinted with permission from [57] 2015 The American Association for the Advancement of Science; (**b**) writing monolithic IC on 2H-MoTe_2_ with a scanning visible light probe, Reprinted with permission from [61] 2018 Springer Nature; (**c**) schematic diagram and (**d**) phase stabilities of monolayer MoTe_2_ under electrostatic gating at constant stress in a capacitor structure, Reprinted with permission from [62] 2016 Springer Nature; (**e**) phase evolution under tensile mechanical deformation, Reprinted with permission from [63] 2014 Springer Nature; (**f**) phase diagram of W alloyed MoTe_2,_ Reprinted with permission from [38] 2017 American Chemical Society.

Laser treatment was reported to induce Te vacancies in MoTe_2_, which thereafter triggered the local phase transition from the 2H phase to 1T′ phase [57,58,59]. Cho et al. used laser-induced phase patterning to fabricate an ohmic 2H–1T′ hetero-phase homojunction that was stable up to 300 °C (Figure 3a) [57]. Not only an ohmic contact between phases but also a patterned phase transfer area could be achieved by this method. By carefully adjusting the laser power and irradiation time, the structural phases of MoTe_2_ could be gradually controlled [60].

Similarly, a scanning visible light probe could be used to directly write electrical circuits onto 2H-MoTe_2_ thin films (Figure 3b) [61]. By creating adatom–vacancy clusters in the host lattice, laser light illumination on patterned metal deposited onto 2H-MoTe_2_ could locally convert the underneath area from an n-type semiconductor to a p-type semiconductor. This doping method could be utilized to assemble both n- and p-doped channels in the same atomic plane at the submicrometer scale, making it possible to fabricate 2D n–p–n (p–n–p) bipolar junction transistor amplifier arrays as well as radial p–n photovoltaic cells.

Electrostatic gating was expected to be a flexible and viable means of phase transition in MoTe_2_. Li et al. predicted the structural semiconductor-to-semimetal phase transition in monolayer MoTe_2_ induced by electrostatic gating [62]. In order to observe 2H–1T′ phase transition in undoped monolayer MoTe_2_ under the constant-stress circumstance, a surface charge density of <−0.04 e or >0.09 e per formula unit was found to be required. Employing a capacitor structure (Figure 3c), a gate voltage of 2–4 V was required to dynamically control the 2H–1T′ phase transition in monolayer MoTe_2_ (Figure 3d). In addition, alloying was predicted to effectively reduce the transition gate voltage—for example, down to 0.3–1 V for Mo_x_W_1−x_Te_2_ monolayers.

As is mentioned above, lattice distortion can also lead to structural transition in MoTe_2_. Duerloo et al. predicted that mechanical deformations could induce phase transition between a semiconducting and a metallic crystal structure in Mo- and W-dichalcogenide monolayers (Figure 3e), and identified that, under uniaxial conditions at room temperature, MoTe_2_ required tensile strains ranging from 0.3 to 3% to have phase transition [63]. Subsequently, a room-temperature 2H–1T′ transition in a MoTe_2_ thin film induced experimentally by a tensile strain of 0.2% was reported [64]. A 2H-MoTe_2_ thin film was transferred to a patterned Si/SiO_2_ substrate with cavities of different diameters, and the researchers then introduced tensile strain using an atomic force microscope (AFM) tip. The supported area without strain remained semiconducting, the suspended area exhibited metallic properties, and the peripheral area displayed both 1T′ and 2H Raman signals. After the release of the strain, the semiconductor–metal phase transition at room temperature was found to be reversible under ambient conditions, which was attributed to the large volume change and small latent heat during the phase transition.

Chalcogen alloying was first introduced to assist electrostatic gating by significantly reducing the transition gate voltage, but, later, alloying alone was found to effectively control the phases of MoTe_2_. Duerloo et al. predicted the structural phase transitions of alloyed MoTe_2_-WTe_2_ monolayers with phase transition temperatures ranging from 0 to 933 K, and mapped its temperature–composition phase diagrams by DFT calculations [65]. Zhang et al. investigated the effect of electrostatic charge injection on the phase stability of monolayer MoTe_2_ and WTe_2_, as well as the effect of transition metal alloying on the phase stability of the H and the T′ phases in monolayer W_x_Mo_1−x_Te_2_ using DFT calculations [66]. Rhodes et al. successfully engineered the phases of MoTe_2_ through W substitution, and plotted the phase diagram of the Mo_1−*x*_W*_x_*Te_2_ solid solution (Figure 3f), which exhibited a semiconducting to semi-metallic transition as a function of x [38]. A small critical W concentration x_c_ ~ 8% was reported to stabilize the γ-phase, which possessed a Fermi surface near that of WTe_2_ at room temperature. The effect of chalcogen alloying on the energy difference and stability between the H and T′ phases in monolayer MoTe_2_ was then studied by DFT calculations [67]. Seven MoTe_2−*x*_X*_x_* alloys (X = N, P, Sb, F, Br, I, and Se) at three concentrations were investigated, and the energy difference between the H and T′ phases was found to be reliant on the chemistry, size, and concentration of the dopants. The p-type and n-type dopants were reported to contribute to defect states atop the valence band and states at the Fermi level of MoTe_2_, respectively.

Li^+^ intercalation was predicted to be possible by DFT calculations to induce phase transition in MoTe_2_ by intercalating Li^+^ ions energetically stable on the 1T′ phase [68]. Utilizing a convection-assisted Li^+^ intercalation method, Eshete et al. converted the surfaces of single-crystalline 2H-MoTe_2_ into the 1T′ phase, and fabricated a vertical 1T′–2H–1T′ MoTe_2_ homojunction [69]. Such a vertical hetero-phase junction was expected to allow active charge transfer and provide numerous catalytic active sites, exhibiting great potential in electrochemical catalysis applications.

## 4. Applications of 2D MoTe_2_ Hetero-Phase Homojunctions

### 4.1. Electronic Devices

Transistors with ohmic contacts have always been in great demand in the semiconductor industry due to their high performance and low power consumption [70,71]. However, the external metal electrodes in transistors tend to induce not only high SBH, resulting from Fermi-level pinning, but also impurities and defects owing to the multi-step procedures of device fabrication, which leads to severe deterioration in device performance [72]. Two-dimensional MoTe_2_ hetero-phase homojunctions provide a solution to these problems. Owing to the small energy barrier between the metallic 1T′ phase and semiconducting 2H phase, the coexistence of the two phases is attainable in transistors as electrodes and channels, respectively. If covalently bonded and atomically coherent, the hetero-phase interface in MoTe_2_ is anticipated to possess an ultralow SBH [16], which incidentally avoids contamination introduced by external metal contacts and unnecessary film depositions. Thus, there are high hopes for 2D MoTe_2_ hetero-phase homojunctions in applications involving electronic devices.

#### 4.1.1. Transistors

**Figure 4:**
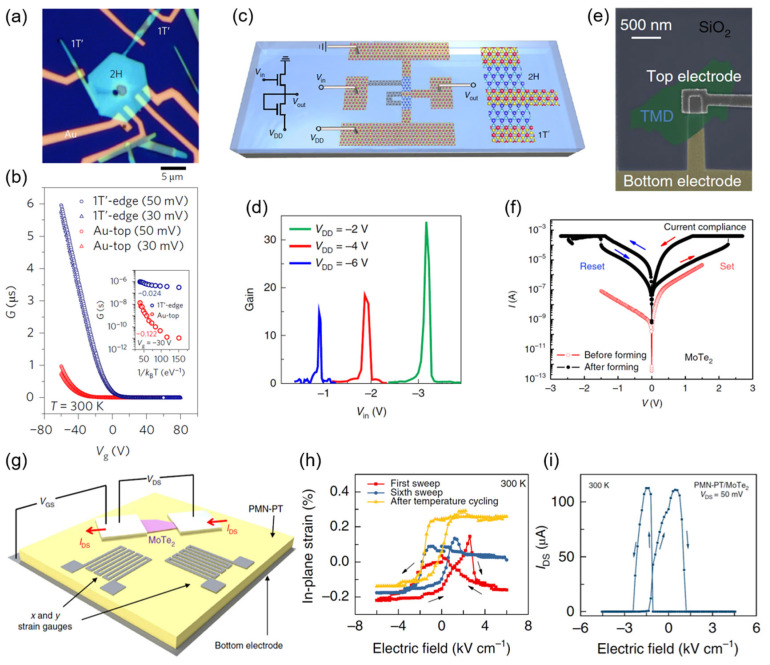
Applications of MoTe_2_ hetero-phase homojunctions in electronic devices. (**a**) Optical microscope image and (**b**) transfer characteristics of a coplanar contacted 1T′–2H-MoTe_2_ transistor [16]; (**c**) schematic diagram and (**d**) signal gains of a hetero-phase MoTe_2_ inverter, Reprinted with permission from [15] 2019 Springer Nature; (**e**) SEM images and (**f**) *I–V* characteristics of a vertical MoTe_2_ RRAM device, Reprinted with permission from [29] 2018 Springer Nature; (**g**) schematic diagram, (**h**) measured strain curves, and (**i**) strain-induced operation of a ferroelectric strain MoTe_2_ FET, Reprinted with permission from [74] 2019 Springer Nature.

Transistors are the basic components of IC, where 2D MoTe_2_ hetero-phase homojunctions have found their way towards high performance. Sung et al. found that the coplanar contacted 1T′–2H MoTe_2_ FETs outperformed conventional Au top-contacted FETs, with significantly higher gate tunability and on current (Figure 4a,b) [16]. By analyzing energy band diagram models and experimental results of the temperature-dependent conductance of FETs at various gate voltages V_g_, they found an ultralow SBH (~25 meV) of the atomically coherent 1T′–2H MoTe_2_ coplanar contact. Similar experimental results in lateral polymorphic MoTe_2_ homojunction FETs fabricated by flux-controlled phase engineering were reported [15,54,73].

The mechanism of the reduced contact resistance in monolayer coplanar polymorphic MX_2_ (MoS_2_, WS_2_, MoSe_2_, WSe_2_, MoTe_2_) FETs with the 2H phase as channels and 1T′ phase as electrodes was demonstrated via ab initio quantum transport simulations [75]. MIGS at the interface was revealed to penetrate the Schottky barrier and connect the valence/conduction band of the channel and the electrodes, consequently leading to a tunable and lower effective SBH and an equivalent ohmic contact under a proper gate voltage. The electronic and contact properties of twelve interface structures of monolayer coplanar 2H–1T′-MoTe_2_ interfaces were investigated using first-principles calculations [76]. The two most energy-favored structures were named as (0°, 30°) and (0°, −30°), and the former was predicted to possess a small SBH, while the Fermi level of the latter was anticipated to be located near the midgap.

Taking advantage of the ultralow contact resistance in 2D MoTe_2_ hetero-phase homojunctions, various applications based on polymorphic MoTe_2_ transistors were exploited—for example, MoTe_2_ radiofrequency (RF) transistors with excellent air stability and a remarkable cutoff frequency (320 MHz with a 5 μm gate length), an ultrashort-gate-length 1T′–2H-MoTe_2_ FET based on a CNT gate with a small subthreshold swing (~73 mV dec^−1^) and a high on/off current ratio (~10^5^), and three-dimensional IC by assembling layers of phase-patterned MoTe_2_ [15].

#### 4.1.2. Logical Devices

By tellurizing patterned different precursors, arrays of hetero-phase MoTe_2_ logic inverters were prepared, which exhibited excellent logic-level conservation, good air stability, and eligibility for IC composed of multiple cascaded inverters (Figure 4c,d) [15]. Under the electric-field-induced strain generated by a ferroelectric substrate, Hou et al. observed the reversible phase transition between few-layer 1T′ and 2H-MoTe_2_ in an FET geometry [73]. This new strategy for transistor switching circumvented the problems of static and dynamic power consumption in conventional FETs. They achieved large nonvolatile changes in channel conductivity between two states at room temperature (Figure 4g–i), indicating the great potential of the sub-nanosecond low-power non-volatile strain switching strategy for applications such as logic and memory devices.

#### 4.1.3. Memory Devices

In vertical 2H-MoTe_2_/Mo_1−x_W_x_Te_2_ resistive random access memory (RRAM) devices, Zhang et al. reported an electric-field-induced phase transition from 2H to 2H_d_ (a distorted transient structure) and the T_d_ phase [29]. They achieved ultrafast, reproducible, resistive switching with on/off current ratios of 10^6^ and programming currents lower than 1 μA in a selectorless RRAM architecture utilizing an Al_2_O_3_/MoTe_2_ stack (Figure 4e,f).

#### 4.1.4. Capacitors

Mixed-dimensional MoTe_2_ structures have shown impressive potential in energy conversion applications. A supercapacitor based on an integrated structure of few-layer 2H-MoTe_2_ and 1T′-Mo_6_Te_6_ nanoplates (NPs) was reported to exhibit enhanced areal capacitance (1542 mF cm^−2^ at 10 mV s^−1^) compared with a transferred monolayer mixed-dimensional electrode, as well as a high energy density (140.36 mW cm^−2^ at 4 mA) and excellent electrochemical stability (96%) [77]. This was attributed to the synergistic effect of 1D Mo_6_Te_6_ NPs and 2D MoTe_2_ layers in the integrated structure, which provided a large surface area and highly conductive pathway, enabling K^+^ ions to diffuse effectively and favoring rapid reversible redox reactions.

### 4.2. Optoelectronic Devices

As is mentioned above, few-layer 2H-MoTe_2_ possesses an indirect bandgap of 0.8 eV, which is suitable for near-infrared (NIR) photodetection [78]. The photogating effect was considered to dominate the photocurrent generation in few-layer 2H-MoTe_2_-based photodetectors and the detailed mechanism was demonstrated as follows: after the generation of electron–hole pairs under irradiation, the charged trap states in the channel acted as a local floating gate and induced more electrons by trapping holes, leading to effective tuning of the channel conductance [79,80].

**Figure 5:**
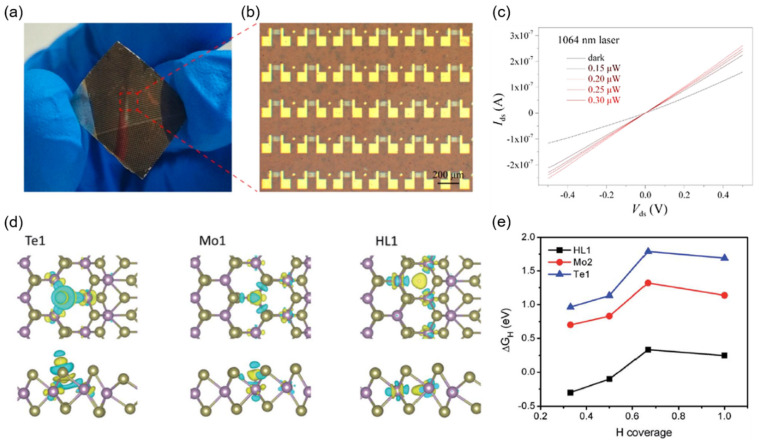
Applications of MoTe_2_ hetero-phase homojunctions in optoelectronic devices and electrocatalysis. (**a**) Macroscopic photograph, (**b**) magnified optical microscope image, and (**c**) *I*–*V* curves of hetero-phase MoTe_2_ photodetectors, Reprinted with permission from [54] 2019 American Chemical Society; (**d**) charge distribution contour plots of hydrogen adsorption sites at the MoTe_2_ phase boundary, Reprinted with permission from [82] 2013 Royal Society of Chemistry; (**e**) plot of Gibbs free energy versus hydrogen coverage for hydrogen adsorption sites, Reprinted with permission from [82] 2013 Royal Society of Chemistry.

For MoTe_2_ hetero-phase homojunctions, photodetectors with both lateral and vertical structures were reported. Via a two-step patterned CVD growth and transfer method, Xu et al. fabricated a lateral 1T′–2H–1T′-MoTe_2_ hetero-phase FET array on a flexible polyimide substrate [54]. They measured the NIR photoresponsivity of the hetero-phase photodetector under different incident light powers and showed a high NIR photoresponsivity of ~1.02 A/W (Figure 5a–c). Lin et al. constructed a vertical 1T′–2H-MoTe_2_ hetero-phase photodetectors and compared the optical response properties to that without inserting 1T′-MoTe_2_ interlayer contact [81].

### 4.3. Electrocatalysis Materials

Furthermore, 1T′-MoTe_2_ was reported to have significant potential in electrocatalysis. Resulting from the adsorption of H atoms onto Te sites on the surface, 1T′-MoTe_2_ exhibited a rapid and reversible activation process where the overpotential required to maintain a certain current density decreased significantly when held at cathodic bias [83].

Chen et al. illustrated that 2H–1T′ phase boundaries could effectively activate the basal plane of monolayer MoTe_2_ for enhanced hydrogen evolution reaction (HER) performance [82]. They investigated, by first-principles calculations, the structural and energetics stabilities of possible configurations of the 2H–1T′ phase boundary, including Te, Mo, and hollow sites, which were identified as possible catalytic centers for the HER at energetically stable phase boundaries (Figure 5d). In particular, the hollow sites newly induced by phase boundaries exhibited comparable Gibbs free energy to that of Pt near the thermoneutral value for H_2_ adsorption (Figure 5e), which was due to the unique electronic structures and local geometries of H_2_ adsorption at phase boundaries.

## 5. Summary and Prospects

In this short review, we summarized the recent progress in 2D MoTe_2_ hetero-phase homojunctions. We first briefly introduced the properties of three phases of MoTe_2_, namely the 2H, 1T′, and T_d_ phases, and then illustrated two strategies, direct synthesis and post-processing, to construct 2D MoTe_2_ hetero-phase homojunctions. We also enumerated the applications of 2D MoTe_2_ hetero-phase homojunctions in electronics, optoelectronics, and electrocatalysis.

Despite the fascinating prospects, 2D MoTe_2_ hetero-phase homojunctions are confronted with several challenges. First, the synthesis and characterization of monolayer MoTe_2_ hetero-phase homojunctions remain to be accomplished, which requires precise control of thickness and careful protection from degradation. Second, to be compatible with the semiconductor industry, facile methods of constructing large-scale patterned MoTe_2_ hetero-phase homojunction arrays are in demand. Lastly, MoTe_2_ hetero-phase homojunctions involving the T_d_ phase and other transient phases beyond need to be further explored.

Various challenges notwithstanding, 2D MoTe_2_ hetero-phase homojunctions are expected to be a stepping stone to super-large-scale IC with extremely high integrity based on pure 2D materials, a fabulous platform for emerging physics research, as well as a promising candidate for future applications.

## Data Availability

Not applicable.
